# Alcohol consumption and lower risk of cardiovascular and all-cause mortality: the impact of accounting for familial factors in twins

**DOI:** 10.1017/S0033291722000812

**Published:** 2023-07

**Authors:** Eivind Ystrom, Eirik Degerud, Martin Tesli, Anne Høye, Ted Reichborn-Kjennerud, Øyvind Næss

**Affiliations:** 1 Norwegian Institute of Public Health, P.O. box 222 Skøyen, 0213 Oslo, Norway; 2 PROMENTA Research Center, Department of Psychology, University of Oslo, P.O. box 1094 Blindern, 0317 Oslo, Norway; 3 NORMENT, Division of Mental Health and Addiction, Oslo University Hospital, Oslo, Norway; 4 Department of Clinical Medicine, UiT – The Arctic University of Norway, Tromsø, Norway; 5 Division of Mental Health and Substance Abuse, University Hospital of North Norway, Tromsø, Norway; 6 Center for Clinical Documentation and Evaluation (SKDE), Tromsø, Norway; 7 Institute of Clinical Medicine, University of Oslo, P.O. box 1170, Blindern, 0318 Oslo, Norway; 8 Institute of Health and Society, University of Oslo, P.O. box 1130, Blindern, 0318 Oslo, Norway

**Keywords:** Alcohol, cardiovascular, genetic, mortality, twin study

## Abstract

**Background:**

A moderate to high alcohol consumption is associated with a lower risk of cardiovascular disease (CVD) mortality in comparison with low consumption. The mechanisms underlying this association are not clear and have been suggested to be caused by residual confounding. The main objective of this study was to separate the familial and individual risk for CVD mortality and all-cause mortality related to alcohol consumption. This will be done by estimating the risk for CVD mortality and all-cause mortality in twin pairs discordant for alcohol consumption.

**Methods:**

Alcohol consumption was assessed at two time points using self-report questionnaires in the Norwegian Twin Registry. Data on CVD mortality was obtained from the Norwegian Cause of Death Registry. Exposure–outcome associations for all-cause mortality and mortality due to other causes than CVD were estimated for comparison.

**Results:**

Coming from a family with moderate to high alcohol consumption was protective against cardiovascular death (HR = 0.54, 95% CI 0.65–0.83). Moderate and high alcohol consumption levels were associated with a slightly increased risk of CVD mortality at the individual level (HR = 1.33, 95% CI 1.02–1.73). There was no association between alcohol consumption and all-cause mortality both at the familial nor at the individual level.

**Conclusions:**

The protective association of moderate to high alcohol consumption with a lower risk of CVD mortality was accounted for by familial factors in this study of twins. Early life genetic and environmental familial factors may mask an absence of health effect of moderate to high alcohol consumption on cardiovascular mortality.

## Introduction

In comparison to abstinence, moderate alcohol consumption is associated with a reduced risk of death due to fatal and non-fatal cardiovascular diseases (CVDs). This reduced risk has been robust to adjustment from other measured cardiovascular risk factors. Because CVD is a major contributor to disease burden and mortality, a reduced risk for CVD also propagates into a lower risk of all-cause mortality (Corrao, Rubbiati, Bagnardi, Zambon, & Poikolainen, 2000; Roerecke & Rehm, 2012; Ronksley, Brien, Turner, Mukamal, & Ghali, 2011). This uneven association between alcohol consumption and CVD forms a ‘J-shaped’ risk curve, which has been found in a wide variety of populations, and moderate drinkers have a 25% lower risk for CVD mortality compared to abstainers (Ronksley et al., 2011). Several harmful and protective effects of alcohol on the cardiovascular system have been suggested to create this non-linear relationship between alcohol and CVD (Brien, Ronksley, Turner, Mukamal, & Ghali, 2011; Krenz & Korthuis, 2012).

However, concerns have been raised as to whether the seemingly protective effect of moderate consumption reflects a causal relationship or is the result of confounding due to variables that are not measured. It would be unethical to randomly assign people to high consumption of alcohol to assess their mortality, and quasi experimental designs are therefore needed to investigate a possible causal link between moderate alcohol consumption and reduced CVD mortality. One such design is Mendelian randomization. People are randomly assigned genotypes during meiosis, and genes related to alcohol consumption but not to confounders can hence be used in a natural experiment where the causal effect is assessed through instrumental variable analysis, wherein people are randomly assigned risk genotypes (Davey Smith & Hemani, 2014). Carriers of the A allele of the alcohol dehydrogenase 1B gene drink less alcohol and have less risk of CVD as suggested by Holmes et al. (2014). The authors of this study concluded that even moderate drinkers would have reduced risk for CVD by reducing alcohol consumption. Also using Mendelian randomization, others have concluded that the association between moderate drinking and low risk for CVD is most likely non-causal (Millwood et al., 2019). Mendelian randomization studies rely on using genetic instruments to cover a complex relationship – the J-shaped form – between different levels of alcohol consumption and CVD risk, but the method typically assumes a linear per-allele effect. The mechanisms involved in explaining the difference in risk between abstainers and moderate consumers may not be the same as the ones involved in explaining the difference between moderate and high consumers (Roerecke & Rehm, 2015).

Confounding may arise due to profound differences between people reporting abstinence or low consumption compared to moderate consumption which the genetic instrumental approaches so far have not been able to pick up. For example, alcohol abstainers have more stressful life events, introverted personality, and risk for anxiety and depression (Rodgers et al., 2000). Studies have shown that alcohol consumption and CVD risk factors cluster in families, which might suggest that the relation between alcohol consumption in adulthood and CVD mortality risk may be affected by family-level confounding. This important public health issue would benefit from causal analysis which deliberately takes a wide range of familial risk into account (Kaprio, 2015).

Alcohol consumption has been found to be substantially dependent on familial factors such as environments shared between siblings in the same household, and genetic factors. Heritability estimates for the underlying risk of high alcohol consumption abuse have been found to range from 50% to 70% (Ystrom, Kendler, & Reichborn-Kjennerud, 2014). Similarly, familial clustering of CVD mortality risk is well known in clinical practice and in numerous studies (Polderman et al., 2015). Likewise, genetic factors are associated with risk factors for CVD (de Oliveira, Pereira, de Andrade, Soler, & Krieger, 2008; Knuiman, Divitini, Welborn, & Bartholomew, 1996; Pilia et al., 2006) and CVD events (Marenberg, Risch, Berkman, Floderus, & de Faire, 1994; Polderman et al., 2015).

Monozygotic twins share both genome and household environmental factors in childhood. Therefore, if there is a difference in CVD mortality between monozygotic twins discordant in drinking, it cannot be due to genetic or familial environmental effects. The protective effect of moderate alcohol consumption on CVD mortality could therefore be due to familial risk in early life, and genetic factors could be influencing both the exposure and the outcome. In fact, if the early life familial confounding is strong enough, it could mask lack of or even a detrimental effect of moderate alcohol consumption on CVD mortality. Such a notion fits well with the results in a study by Holmes et al. wherein individuals having a genetic variant associated with low alcohol consumption had lower risk of CVD mortality.

We are not aware of any previous study estimating the protective effect of moderate alcohol consumption on CVD risk within twin pairs. A null finding within twin pairs would be a strong argument against any such protective effect in the general population.

By linking a national twin registry to a cause of death registry, we aim to estimate the causal effect of moderate alcohol consumption on CVD mortality, all-cause mortality, and non-CVD mortality within twin pairs.

## Methods

### Participants

The sample comprises 14 496 individuals from 5520 complete same sex twin pairs (1101 monozygotic male, 1326 dizygotic male, 1443 monozygotic female, and 1650 dizygotic female) and 3456 single responders born 1915–1960 from Panel II in the Norwegian Twin Registry where both were alive at age 20 (Nilsen, Brandt, Magnus, & Harris, 2012). Zygosity was determined by questionnaire (Q1), and subsequently by genetic marker analysis for a sub-sample (Nilsen et al., 2012). Twins responding to Q1 were sent a health questionnaire, including questions on alcohol consumption, in 1978–1982 (Q2) and again in 1990–1998 (Q3). Our sample comprises individuals responding to either or both of these questionnaires.

### Alcohol exposure

In Q2 the responders were asked if they were drinking beer (bottle), wine (glass), or liquor (glass) using response categories (1) no, (2) less than one unit per day, (3) 1–2 units per day, or more than two units per day. In Q3 they were asked if they had drunk alcohol in the last year; how often they drank alcohol; number of beer, wine, and liquor units per week; and how often they drank five or more units of alcohol. To combine all the measures from the two questionnaires into an alcohol continuum score (Krueger et al., 2004) we subjected all the items to a graded response item response theory (IRT) analysis.

### Cardiovascular mortality

The Norwegian Cause of Death Registry provided outcome data on causes of death using the ninth and tenth revision of the International Classification of Diseases (ICD). The primary outcomes were CVD mortality (1990–1995: ICD-9 390–459; 1996–2014: ICD-10 I00–I99) and all causes of death. The registry is almost exclusively based on certificates filled out by on-site medical doctors, and in the few cases in which autopsies are performed, 32% of deaths are reclassified over major ICD-10 chapters (Alfsen & Maehlen, 2012).

### Statistical analyses

We aggregated indicators of alcohol consumption by applying a graded response model (GRM) IRT model (Samejima, 1968; Thissen & Steinberg, 1986). The GRM-IRT is a logistic model where, based on their response pattern, each individual is placed on a standard normal continuum. The GRM-IRT has two parameters for each observed variable, and each variable is characterized by their ‘difficulty’ (i.e. severity) and ‘discriminability’. While the difficulty parameter, or threshold, localizes each item on the standard normal continuum, the discriminability parameter, or slope, represents how well an item can reliably differentiate individuals on this continuum. We saved out empirical Bayes means as IRT-scores and divided these into quintiles.

We estimated the heritability of cause-specific mortality by using a logistic generalized structural equation model with a frailty parameter for between twin effects. The between twin effect was estimated separately for monozygotic and dizygotic twins. By genetic theory we constrained the between monozygotic twin effect to be all additive genetic, non-additive genetic, familial environmental (or shared environmental) effects. Since dizygotic twins share 50% of their segregating genes and 25% of non-additive genetic effects, we constrained the between dizygotic twin effect to be 0.5 for additive genetic effects and 0.25 non-additive genetic effects. As monozygotic twins, dizygotic twins share all familial environmental effects, we constrained the between environmental effect for both monozygotic and dizygotic twins unity. We estimated the individual-specific environmental effect by subtracting the between twin effect from the total observed variance [i.e. all between effects + 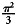 (variance of the logistic distribution)].

To estimate the association between alcohol consumption and CVD mortality, we used a mixed-effects Weibull proportional hazard regression model with individuals on level 1, zygotes on level 2, and pregnancies on level 3 (Rabe-Hesketh, Skrondal, & Gjessing, 2008). Data were censored either at time of death or at the end of the study. Since monozygotic pairs share 100% of their genome, we assumed that the cluster mean fixed effect represented 100% of the genetic covariance (Rabe-Hesketh et al., 2008). We centered the alcohol consumption score on the cluster mean, and assumed that the within pair fixed effects for monozygotic twins represented environmental effects. What is more, we assumed that the within pair fixed effect for dizygotic pairs represented the within monozygotic pair effect plus half of the genetic effect. Since dizygotic pairs share 50% of their genome, we furthermore assumed that the group mean fixed effect for dizygotic pairs represented 50% of the genetic covariance. We did the generalized structural equation modeling in Mplus 8.0 and all other analyses in STATA 15.1.

## Results

### All-cause and cardiovascular mortality

At the end of observation, we found that 3633 of the 14 496 individuals had died of any cause. Among these, 1207 (33%) had died of CVD, and 2426 (67%) had died of other causes than CVD. The average age of death for all-cause, CVD, and non-CVD was 72.5 (range 24–100 years; s.d. = 13.3), 74.9 (range 32–99 years; s.d. = 12.1), and 71.3 (range 24–97 years; s.d. = 13.7), respectively.

### Item response theory modeling of the alcohol consumption continuum

We present the detailed results from the GRM-IRT analyses in online Supplementary S1. All the items proved to have discriminative power with different levels of severity on the alcohol consumption continuum. What is more, the alcohol continuum score proved to have a substantial degree of information (i.e. reliability) from −1.5 to 4 s.d. For secondary analyses we calculated factor scores (empirical Bayes means). For the survival analyses, we chose to stratify the score into quintiles. Across the quintiles, the alcohol score average ± standard deviation was −1.35 ± 0.31, −0.58 ± 0.23, 0.08 ± 0.14, 0.68 ± 0.16, and 1.25 ± 0.40; and the *α* reliability was 0.66, 0.84, 0.82, 0.75, 0.82; respectively.

In Table 1, we present the alcohol consumption pattern within each quintile. Quintile one was characterized by almost total abstinence from alcohol consumption. Quintile two was characterized by non-abstinence, but infrequent alcohol consumption, e.g. 68.4% drank alcohol less than once a month. In Quintile three the majority (89.9%) consumed alcohol 2–3 times per month or less, but did to a much larger extent drink different types of alcohol beverages than people in quintile 2. Quintile four was characterized by more frequent drinking and 77.7% drinking five or more units 1–4 times per year or more. Quintile five was characterized by 90.9% drinking alcohol once per week or more and 59.9% reported frequent binge drinking, defined by a consumption of five or more units of alcohol on a single occasion at least once per month.

**Table 1.** Alcohol use behavior per quintile of alcohol use in 14 496 twins

	Quintile *N* %				
	1	2	3	4	5
	3343	2485	2870	3982	1816
	23.1%	17.1%	19.8%	27.5%	12.5%
*Questionnaire 2*					
Do you drink beer?					
No (%)	99.6	85.3	37.9	5.1	3.8
Less than one unit per day (%)	0.4	14.6	60.8	93.2	77.9
1–2 units per day or more than two units per day (%)	0.0	0.0	0.9	1.6	14.0
More than one unit per day (%)	0.0	0.0	0.3	0.1	4.3
Responses (*n*)	3171	2237	2656	3833	1604
Non-response (*n*)	172	248	214	149	212
Do you drink wine?					
No (%)	99.4	77.3	38.1	7.2	9.0
Less than one unit per day (%)	0.6	22.2	61.9	92.5	86.2
1–2 units per day or more than two units per day (%)	0.0	0.5	0.0	0.2	4.4
More than one unit per day (%)	0.0	0.1	0.0	0.1	0.4
Responses (*n*)	3171	2237	2656	3833	1604
Non-response (*n*)	172	248	214	149	212
Do you drink liquor?					
No	99.6	71.8	19.7	2.58	1.2
Less than one unit per day	0.4	28.3	79.7	97.3	88.8
1–2 units per day or more than two units per day	0.0	0.0	0.6	0.1	8.4
More than one unit per day	0.0	0.0	0.0	0.0	1.6
Responses (*n*)	3171	2237	2656	3833	1604
Non-response (*n*)	172	248	214	149	212
*Questionnaire 3*					
Have you drunk alcohol in the last year?					
Yes (%)	0.9	95.1	99.5	99.9	99.9
Responses (*n*)	987	1606	1501	1473	1428
Non-response (*n*)	2356	879	1369	2509	388
How often do you drink alcohol?					
Never	88.9	3.5	0.5	0.0	0.0
Less than once a month	11.1	68.4	30.1	2.3	0.0
About once a month	0.0	16.0	32.0	11.5	0.5
2–3 times per month	0.0	10.0	27.3	48.2	9.6
About once per week	0.0	1.7	8.6	34.0	50.9
2–4 times per day	0.0	0.3	1.3	3.6	33.7
Every day or almost every day	0.0	0.0	0.3	0.3	5.3
Responses (*n*)	902	1709	1610	1546	1486
Non-response (*n*)	2441	776	1260	2436	330
How many bottles of beer did you drink last week?					
0	99.24	91.0	75.1	47.0	21.8
1	0.76	5.5	14.5	26.7	17.6
2	0.0	2.0	6.0	14.9	20.0
3	0.0	0.7	1.9	5.0	10.3
4	0.0	0.2	1.1	2.5	9.3
5	0.0	0.1	0.3	1.5	4.7
6	0.0	0.1	0.3	0.9	5.3
7	0.0	0.1	0.1	0.7	3.4
8	0.0	0.3	0.4	0.6	4.1
≥9	0.0	0.1	0.2	0.4	3.5
Responses (*n*)	395	1516	1478	1463	1442
Non-response (*n*)	2948	969	1392	2519	374
How many glasses of wine did you drink last week?					
0	98.7	83.5	69.2	49.5	29.5
1	0.8	7.5	10.3	10.7	5.9
2	0.26	5.7	12.8	22.5	18.0
3	0.26	1.8	3.7	8.2	13.0
4	0.0	0.7	2.1	5.0	13.2
5	0.0	0.3	0.7	1.3	5.5
6	0.0	0.1	0.7	1.3	6.1
7	0.0	0.1	0.1	0.6	3.8
8	0.0	0.1	0.2	0.4	2.7
≥9	0.0	0.1	0.1	0.4	2.3
Responses (*n*)	392	1500	1480	1432	1368
Non-response (*n*)	2951	985	2870	2550	448
How many drinks of liquor did you drink last week?					
0	100.0	91.8	79.4	60.6	36.1
1	0.0	4.6	10.6	15.3	13.6
2	0.0	2.4	5.3	12.9	18.2
3	0.0	0.7	2.4	4.3	10.6
4	0.0	0.1	1.0	3.8	7.9
5	0.0	0.0	0.6	1.4	4.7
6	0.0	0.1	0.3	0.4	3.1
7	0.0	0.1	0.1	0.6	2.2
8	0.0	0.1	0.3	0.5	1.8
≥9	0.0	0.1	0.1	0.35	1.8
Responses (*n*)	381	1488	1459	1432	1402
Non-response (*n*)	2962	997	2870	2550	414
How often do you drink five units of alcohol?					
Never	97.9	59.8	36.6	22.3	7.1
1–4 times per year	2.0	32.9	40.5	33.0	15.5
5–10 times per year	0.1	6.2	15.7	22.6	17.7
Once per month	0.0	1.1	4.3	12.2	17.6
2–3 times per month	0.0	0.1	1.2	7.0	17.6
Once per week	0.0	0.0	0.9	2.6	18.9
2–4 times per week	0.0	0.0	0.0	0.3	4.9
Almost every day or every day	0.0	0.0	0.0	0.0	0.9
Responses (*n*)	980	1696	1.613	1530	1481
Non-response (*n*)	2363	789	1257	2452	335

### Heritability of alcohol consumption and cause-specific cardiovascular disease mortality

We found that factors for alcohol consumption in the general population could be divided into 31% (95% CI 25–38%) additive genetic factors, 30% (95% CI 25–37%) shared environmental factors, and 39% (95% CI 37–42%) individual-specific environmental factors.

We found CVD mortality to be influenced by additive genetic and non-additive genetic factors. Specifically, we estimated additive genetic, non-additive genetic, and individual specific risk factors to explain 13% (95% CI −0.13 to 38%), 34% (95% CI 7–61%), and 53% (95% CI 47–59%) of variance in risk factors in the general population. We estimated the total, or broad sense, heritability (i.e. all genetic effects) of CVD mortality to be 47% (95% CI 41–53%).

### Alcohol consumption and cause-specific cardiovascular disease mortality: familial and individual risk factors

**Fig. 1.**
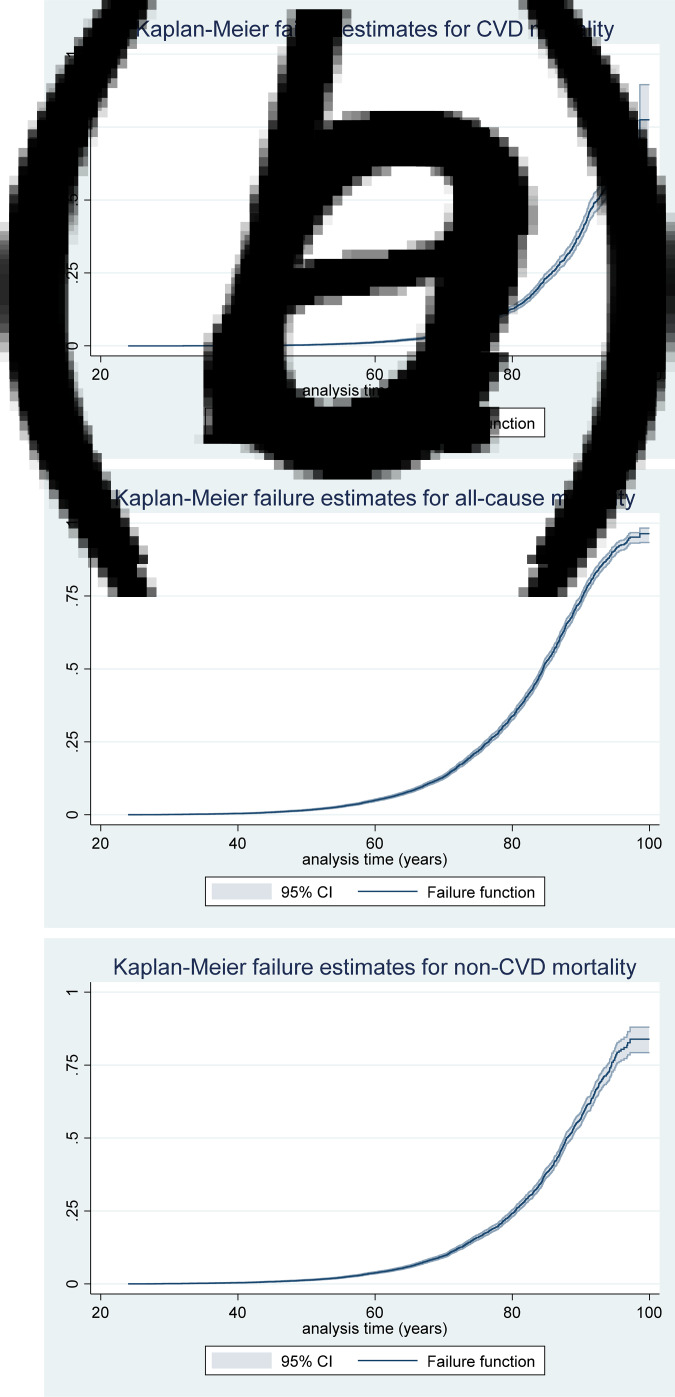
Empirical Kaplan–Meier failure curves in 14 496 twins.

In Fig. 1*a* we present Kaplan–Meier failure curves for CVD mortality. We estimate that 75% of individuals in the population would have died of a CVD by age 100, provided that there were no competing risks. In Fig. 1*b* we present the model-derived cumulative hazard. The model-derived function appears to have a close fit to the empirical data.

We present the between individual (traditional cohort analysis), familial, and individual specific effects of alcohol consumption on CVD mortality in Table 2. In the analysis performed using the study population as a cohort, the risk of CVD death was 30% lower (HR = 0.70; 95% CI 0.61–0.81) in the top two quintiles of alcohol consumption compared to low to moderate consumers (quintile 1–3). Two, the familial risk factors (i.e. genetic factors varying between monozygotic twin pairs) for increasing levels of alcohol consumption are inversely related to CVD mortality. Familial risk for the top two quintiles of alcohol consumption gives a 46% reduction in risk for death due to CVD (HR = 0.54; 95% CI 0.65–0.83). Three, the co-twin control analysis shows that alcohol consumption corresponding to the top two quintiles in the population gives an increase in risk for death from a CVD (HR = 1.33; 95% CI 1.02–1.73) when comparing with low consumption.

**Table 2.** Hazard ratios for cardiovascular-specific mortality following quintiles of alcohol use in 14 496 twins

Quintiles	Cohort (between individuals)		Familial effect (est. between MZ twin pairs)		Individual-specific effects (est. within MZ twin pairs)	
	HR	(95% CI)	HR	(95% CI)	HR	(95% CI)
All levels of consume						
1	1.00	–	1.00	–	1.00	
2	0.73	(0.61–0.87)	0.67	(0.51–0.89)	1.04	(0.74–1.47)
3	0.79	(0.66–0.94)	0.87	(0.67–1.13)	0.98	(0.68–1.40)
4	0.60	(0.49–0.72)	0.48	(0.36–0.63)	1.31	(0.88–1.96)
5	0.61	(0.48–0.77)	0.50	(0.35–0.71)	1.31	(0.80–2.16)
High consumers *v.* low						
1 + 2 + 3	1.00		1.00		1.00	
4 + 5	0.70	(0.61–0.81)	0.54	(0.65–0.83)	1.33	(1.02–1.73)

In Table 3, we present the corresponding analyses for all-cause mortality. One, in the cohort analyses, we found high alcohol consumption to be marginally protective for all-cause mortality (HR = 0.92; 95% CI 0.86–0.99). Two, the familial risk factors for increasing levels of alcohol consumption are inversely related to all-cause mortality (HR = 0.88; 95% CI 0.79–0.98). Three, the co-twin control analysis shows that alcohol consumption corresponding to the top two quintiles in the population is not protective for all-cause mortality (HR = 1.09; 0.96–1.25).

**Table 3.** Hazard ratios for all-cause mortality following quintiles of alcohol use in 14 496 twins

Quintiles	Cohort (between individuals)		Familial effect (est. between MZ twin pairs)		Individual-specific effects (est. within MZ twin pairs)	
	HR	(95% CI)	HR	(95% CI)	HR	(95% CI)
All levels of consume						
1	1.00	–	1.00	–	1.00	
2	0.88	(0.80–0.97)	0.87	(0.75–1.00)	0.95	(0.78–1.16)
3	0.93	(0.84–1.03)	0.97	(0.85–1.12)	0.91	(0.74–1.11)
4	0.87	(0.79–0.95)	0.85	(0.75–0.97)	1.00	(0.81–1.25)
5	0.89	(0.59–0.67)	0.85	(0.71–1.01)	1.08	(0.83–1.41)
High consumers *v.* low						
1 + 2 + 3	1.00		1.00		1.00	
4 + 5	0.92	(0.86–0.99)	0.88	(0.79–0.98)	1.09	(0.96–1.25)

For non-CVD mortality (Table 4), we found no association with high alcohol consumption (HR = 1.05; 0.96–1.15) nor at the familial (HR = 1.08; 0.96–1.23) or within MZ twin pair level (HR = 1.02; 0.86–1.21).

**Table 4.** Hazard ratios for non-cardiovascular mortality following quintiles of alcohol use in 14 496 twins

Quintiles	Cohort (between individuals)		Familial effect (est. between MZ twin pairs)		Individual-specific effects (est. within MZ twin pairs)	
	HR	(95% CI)	HR	(95% CI)	HR	(95% CI)
All levels of consume						
1	1.00	–	1.00	–	1.00	
2	0.97	(0.86–1.10)	0.97	(0.81–1.16)	0.91	(0.71–1.15)
3	1.01	(0.90–1.14)	1.02	(0.86–1.21)	0.87	(0.68–1.12)
4	1.03	(0.93–1.23)	1.09	(0.93–1.28)	0.90	(0.69–1.17)
5	1.07	(0.93–1.23)	1.07	(0.87–1.31)	0.99	(0.72–1.37)
High consumers *v.* low						
1 + 2 + 3	1.00		1.00		1.00	
4 + 5	1.05	(0.96–1.15)	1.08	(0.96–1.23)	1.02	(0.86–1.21)

## Discussion

Increasing alcohol consumption is associated with lower risk of death by CVD in traditional analyses of cohort data, but both alcohol consumption and CVD mortality are influenced by genetic and early life environmental factors. We have shown that these familial factors for high alcohol consumption are protective for CVD mortality, and when the familial effects are adjusted for, the picture is turned upside down and high alcohol consumption gives an increased risk of CVD mortality on the individual level. The same was not evident for all-cause mortality or non-CVD mortality.

Our findings on CVD mortality show that when confounding factors are inverse to a direct effect, any possible causal exposure–outcome effect is suppressed. As an example using intelligence measured at age 18, Degerud et al. found that high alcohol consumption in midlife was associated with high intelligence in mid adulthood (Degerud et al., 2017). Intelligence is substantially heritable (Polderman et al., 2015), and this could indicate that familial factors for alcohol consumption represent a general protective factor related to high education. This is opposite to what was found in a smaller sample of twins (Dai, Mukamal, Krasnow, Swan, & Reed, 2015), where the twin with the highest consumption of alcohol had lower chance of CVD mortality. However, our results indicate that genetic factors are of importance, and Dai et al. (2015) were not able to perform separate analyses for monozygotic and dizygotic twins, leaving residual genetic confounding for the exposure–outcome association (Kaprio, 2015). The current study contributes by separating out the familial effect, and hence identifying a mechanism clouding the alcohol consumption–CVD mortality association. Our findings were in concordance with a large Mendelian randomization instrumental variable study (Holmes et al., 2014), finding that moderate to high consumption leads to an increased risk for CVD. Both genetically informative causal inference approaches hence lean in the same direction, amounting to triangulation of evidence from etiological studies (Lawlor, Tilling, & Davey Smith, 2016).

For all-cause mortality we found alcohol consumption to be moderately protective in the cohort analyses. The main difference was evident when comparing drinkers to the lowest level of alcohol consumption. However, when adjusting for familial factors, there was no protective association between high alcohol consumption and all-cause mortality, which is in line with the findings for CVD mortality. For non-CVD mortality, we did not find an association with familial risk for high alcohol consumption. This rendered the estimated within MZ twin pair alcohol consumption–mortality association close to null.

To date there are 35 studies on the heritability of all-cause mortality (Polderman et al., 2015). In a meta-study, the estimated narrow sense heritability is 29% for males and 25% for females (Polderman et al., 2015). Although the heritability of death from coronary heart disease (CHD) has been estimated using a Swedish twin sample (Zdravkovic et al., 2002), this is the first study estimating the heritability of CVD mortality. We estimated the broad sense heritability to be 47%. However, 72% of these genetic effects were estimated to be attributed to non-additive effects, such as interactions between genetic variants on the same or different loci on the genome. Only 28% were estimated to be due to additive genetic effects. This is similar to the Swedish study on CHD, estimating a broad sense heritability of 59% for males and 39% for females with indications of non-additive genetic effects. There are two reasons our results on non-additive genetic effects are important. One, only additive genetic effects are estimated using molecular genetic data such as single nucleotide polymorphisms, and only such effects are estimated in genome-wide association studies. If these estimates reflect the population values, we would expect to have a great share of ‘missing heritability’ in molecular genetic studies on CVD mortality. Two, non-additive genetic risk is not intergenerationally transmitted from parent to offspring, only additive genetic risk. This means that if our heritability estimates are valid, CVD in parents and grandparents is only partly indicative of familial genetic risk for CVD.

We found familial factors to account for 61% of the risk for alcohol consumption, with approximately equal contribution from additive genetic factors and shared environmental factors. These effects are similar to what was found in a meta-study comprising 405 twin studies on alcohol consumption with familial factors accounting for 63% of the risk (Polderman et al., 2015). However, previous studies on alcohol use disorders among Norwegian twins born a generation later (1967–1979) have found higher genetic effects and lower shared environmental effects (Torvik et al., 2017; Ystrom et al., 2014). It could be that alcohol use disorders are more heritable than alcohol consumption levels in the general population (Ystrom, Reichborn-Kjennerud, Aggen, & Kendler, 2011).

### Limitations

Our study has some limitations. One, although we censored the data at the end of observation time, our sample was by and large still alive. It could be that alcohol consumption is more strongly linked to early-onset CVD, and that the association in an end of life sample would have been smaller. Two, the classical twin design allows for estimating either shared environmental effects or non-additive genetic effects, but not both at the same time. Strong non-additive genetic effects could have masked shared environmental risk factors for CVD mortality, and, conversely, shared environmental effects could have masked non-additive genetic risk factors for alcohol consumption. Three, although the alcohol exposure we used was reliable to several distinctive categories of alcohol consumption, we were not able to quantify the amount in grams. We had limited number of CVD risk factors available in the twin data, thus we could not identify which of these may contribute to familial protective effect of alcohol consumption. Four, although we could adjust for familial factors by design, we could not adjust for unmeasured individual risk factors associated with both alcohol consumption and mortality, such as early-onset psychiatric disorders. Five, we had no information on CVD treatment, and could therefore not adjust for possible individual treatment effects on CVD mortality. Six, we relied on subjective reporting of alcohol consumption. Measurement error could have led to an underestimation of effects. Seven, there is a risk of misclassification of CVD deaths in cause of death registries, due to both unrecognizing actual CVD deaths (e.g. sudden deaths) as well as describing non-cardiovascular deaths cardiovascular. The accuracy of registered CVD deaths in the Norwegian Cause of Death Registry has, however, been shown to be satisfactory (Gulsvik et al., 2011).

## Conclusion

Moderate to high alcohol consumption was associated with a lower risk of CVD as well as all-cause mortality in a traditional cohort design analysis, but when adjusting for familial factors in a twin design, there was not protective association. Rather, alcohol consumption was associated with a higher risk of CVD mortality. This suggests that genetic and early life familial factors are protective against alcohol consumption and have masked the true causal effect of alcohol consumption in traditional cohort analyses.

## Supplementary material

For supplementary material accompanying this paper visit https://doi.org/10.1017/S0033291722000812. click here to view supplementary material
