# Supplementary material for: Alcohol consumption and lower risk of cardiovascular and all-cause mortality: the impact of accounting for familial factors in twins
Source: Psychol Med. 2022 Apr 20;53(9):4130–8. doi: 10.1017/S0033291722000812 (PMC10317821; doi:10.1017/S0033291722000812)
Supplement: Supplementary file 1 [file S0033291722000812sup001.docx]

Online supplement 1

**Supplementary table 1.1. Item response theory model parameters.**

|  |  |  | Label | *Discriminability* | *Difficulty* | *95% CI* | | | | |
| --- | --- | --- | --- | --- | --- | --- | --- | --- | --- | --- |
| **Questionnaire 2** | | |  |  |  |  |  |  |  |  |
|  | *Do you drink beer?* | | beerunQ2 | 2.38 |  | ( | 2.26 | - | 2.50 | ) |
|  |  | Less than one unit per day | beerunQ2>=1 |  | -0.13 | ( | -0.16 | - | -0.09 | ) |
|  |  | 1-2 units per day or more than two units per day | beerunQ2>=2 |  | 2.33 | ( | 2.25 | - | 2.41 | ) |
|  |  | More than one unit per day | beerunQ2=3 |  | 3.10 | ( | 2.98 | - | 3.23 | ) |
|  |  |  |  |  |  |  |  |  |  |  |
|  | *Do you drink wine?* | | wineunQ2 | 2.08 |  | ( | 1.97 | - | 2.20 | ) |
|  |  | Less than one unit per day | wineunQ2>=1 |  | -0.14 | ( | -0.18 | - | -0.10 | ) |
|  |  | 1-2 units per day or more than two units per day | wineunQ2>=2 |  | 3.21 | ( | 3.07 | - | 3.35 | ) |
|  |  | More than one unit per day | wineunQ2=3 |  | 4.31 | ( | 3.99 | - | 4.63 | ) |
|  |  |  |  |  |  |  |  |  |  |  |
|  | *Do you drink liquor?* | | spirunQ2 | 2.97 |  | ( | 2.79 | - | 3.15 | ) |
|  |  | Less than one unit per day | spirunQ2>=1 |  | -0.33 | ( | -0.36 | - | -0.29 | ) |
|  |  | 1-2 units per day or more than two units per day | spirunQ2>=2 |  | 2.55 | ( | 2.46 | - | 2.63 | ) |
|  |  | More than one unit per day | spirunQ2=3 |  | 3.28 | ( | 3.12 | - | 3.45 | ) |
|  |  |  |  |  |  |  |  |  |  |  |
| **Questionnaire 3** | | |  |  |  |  |  |  |  |  |
|  | *Have you drunk alcohol in the last year?* | | drink12Q3 | -3.94 |  | ( | -4.40 | - | -3.47 | ) |
|  |  | Yes | drink12Q3=2 |  | -1.27 | ( | -1.33 | - | -1.21 | ) |
|  |  |  |  |  |  |  |  |  |  |  |
|  | *How often do you drink alcohol?* | | alko1Q3 | 2.57 |  | ( | 2.46 | - | 2.67 | ) |
|  |  | Less than once a month | alko1Q3>=2 |  | -1.54 | ( | -1.62 | - | -1.46 | ) |
|  |  | About one a month | alko1Q3>=3 |  | -0.36 | ( | -0.41 | - | -0.31 | ) |
|  |  | 2-3 times per month | alko1Q3>=4 |  | 0.05 | ( | 0.01 | - | 0.10 | ) |
|  |  | About once per week | alko1Q3>=5 |  | 0.71 | ( | 0.66 | - | 0.76 | ) |
|  |  | 2-4 times per day | alko1Q3>=6 |  | 1.58 | ( | 1.53 | - | 1.63 | ) |
|  |  | Every day or almost every day | alko1Q3>=7 |  | 2.46 | ( | 2.37 | - | 2.54 | ) |
|  |  |  |  |  |  |  |  |  |  |  |
|  | *How many bottles of beer did you drink last week?* | | bottweoQ3 | 1.59 |  | ( | 1.50 | - | 1.69 | ) |
|  |  | 1 | bottweoQ3>=1 |  | 0.57 | ( | 0.52 | - | 0.63 | ) |
|  |  | 2 | bottweoQ3>=2 |  | 1.18 | ( | 1.11 | - | 1.25 | ) |
|  |  | 3 | bottweoQ3>=3 |  | 1.72 | ( | 1.64 | - | 1.81 | ) |
|  |  | 4 | bottweoQ3>=4 |  | 2.04 | ( | 1.94 | - | 2.14 | ) |
|  |  | 5 | bottweoQ3>=5 |  | 2.35 | ( | 2.24 | - | 2.47 | ) |
|  |  | 6 | bottweoQ3>=6 |  | 2.57 | ( | 2.44 | - | 2.70 | ) |
|  |  | 7 | bottweoQ3>=7 |  | 2.86 | ( | 2.71 | - | 3.01 | ) |
|  |  | 8 | bottweoQ3>=8 |  | 3.12 | ( | 2.95 | - | 3.29 | ) |
|  |  | >= 9 | bottweoQ3=9 |  | 3.67 | ( | 3.44 | - | 3.90 | ) |
|  |  |  |  |  |  |  |  |  |  |  |
|  | *How many glasses of wine did you drink last week?* | | glasweoQ3 | 1.24 |  | ( | 1.14 | - | 1.33 | ) |
|  |  | 1 | glasweoQ3>=1 |  | 0.58 | ( | 0.51 | - | 0.65 | ) |
|  |  | 2 | glasweoQ3>=2 |  | 0.94 | ( | 0.86 | - | 1.01 | ) |
|  |  | 3 | glasweoQ3>=3 |  | 1.69 | ( | 1.58 | - | 1.80 | ) |
|  |  | 4 | glasweoQ3>=4 |  | 2.17 | ( | 2.03 | - | 2.31 | ) |
|  |  | 5 | glasweoQ3>=5 |  | 2.74 | ( | 2.56 | - | 2.93 | ) |
|  |  | 6 | glasweoQ3>=6 |  | 3.06 | ( | 2.85 | - | 3.27 | ) |
|  |  | 7 | glasweoQ3>=7 |  | 3.56 | ( | 3.31 | - | 3.81 | ) |
|  |  | 8 | glasweoQ3>=8 |  | 4.04 | ( | 3.74 | - | 4.34 | ) |
|  |  | >= 9 | glasweoQ3=9 |  | 4.69 | ( | 4.30 | - | 5.07 | ) |
|  |  |  |  |  |  |  |  |  |  |  |
|  | *How many drinks of liquor did you drink last week?* | | drinkweoQ3 | 1.30 |  | ( | 1.21 | - | 1.39 | ) |
|  |  | 1 | drinkweoQ3>=1 |  | 0.95 | ( | 0.88 | - | 1.02 | ) |
|  |  | 2 | drinkweoQ3>=2 |  | 1.47 | ( | 1.38 | - | 1.56 | ) |
|  |  | 3 | drinkweoQ3>=3 |  | 2.09 | ( | 1.97 | - | 2.22 | ) |
|  |  | 4 | drinkweoQ3>=4 |  | 2.53 | ( | 2.37 | - | 2.68 | ) |
|  |  | 5 | drinkweoQ3>=5 |  | 3.00 | ( | 2.81 | - | 3.19 | ) |
|  |  | 6 | drinkweoQ3>=6 |  | 3.39 | ( | 3.17 | - | 3.60 | ) |
|  |  | 7 | drinkweoQ3>=7 |  | 3.72 | ( | 3.47 | - | 3.96 | ) |
|  |  | 8 | drinkweoQ3>=8 |  | 4.09 | ( | 3.80 | - | 4.37 | ) |
|  |  | >= 9 | drinkweoQ3=9 |  | 4.70 | ( | 4.33 | - | 5.07 | ) |
|  |  |  |  |  |  |  |  |  |  |  |
|  | *How often do you drink five units of alcohol?* | | excessQ3 | 1.62 |  | ( | 1.54 | - | 1.71 | ) |
|  |  | 1-4 times per year | excessQ3>=2 |  | -0.35 | ( | -0.40 | - | -0.29 | ) |
|  |  | 5-10 times per year | excessQ3>=3 |  | 0.65 | ( | 0.59 | - | 0.70 | ) |
|  |  | Once per month | excessQ3>=4 |  | 1.25 | ( | 1.18 | - | 1.31 | ) |
|  |  | 2-3 times per month | excessQ3>=5 |  | 1.70 | ( | 1.62 | - | 1.78 | ) |
|  |  | Once per week | excessQ3>=6 |  | 2.21 | ( | 2.11 | - | 2.31 | ) |
|  |  | 2-4 times per week | excessQ3>=7 |  | 3.09 | ( | 2.95 | - | 3.23 | ) |
|  |  | Almost every day or every day | excessQ3>=8 |  | 3.57 | ( | 3.39 | - | 3.75 | ) |

**Figures for Item Response Theory modeling of alcohol consumption**

**Boundary Characteristic curves for indicators of the alcohol use continuum**

| **Questionnaire 2** |  |
| --- | --- |
|  |  |
| *Do you drink beer?*  Less than one unit per day - beerunQ2>=1  1-2 units per day or more than two units per day - beerunQ2>=2  More than one unit per day - beerunQ2=3 | *Do you drink wine?*  Less than one unit per day - wineunQ2>=1  1-2 units per day or more than two units per day - wineunQ2>=2  More than one unit per day - wineunQ2=3 |

|  |  |
| --- | --- |
| *Do you drink liquor?*  Less than one unit per day - spirunQ2>=1  1-2 units per day or more than two units per day - spirunQ2>=2  More than one unit per day - spirunQ2=3 |  |

|  |  |
| --- | --- |
|  |  |
| **Questionnaire 3** |  |
|  |  |
| *Have you drunk alcohol in the last year?*  Yes - drink12Q3=2 | *How often do you drink alcohol?*  Less than once a month - alko1Q3>=2  About one a month - alko1Q3>=3  2-3 times per month - alko1Q3>=4  About once per week - alko1Q3>=5  2-4 times per day - alko1Q3>=6  Every day or almost every day - alko1Q3>=7 |
|  |  |
| *How many bottles of beer did you drink last week?*  1 - bottweoQ3>=1  2 - bottweoQ3>=2  3 - bottweoQ3>=3  4 - bottweoQ3>=4  5 - bottweoQ3>=5  6 - bottweoQ3>=6  7 - bottweoQ3>=7  8 - bottweoQ3>=8  >= 9 - bottweoQ3=9 | How many glasses of wine did you drink last week?  1 - glasweoQ3>=1  2 - glasweoQ3>=2  3 - glasweoQ3>=3  4 - glasweoQ3>=4  5 - glasweoQ3>=5  6 - glasweoQ3>=6  7 - glasweoQ3>=7  8 - glasweoQ3>=8  >= 9 - glasweoQ3=9 |
|  |  |
| How many drinks of liquor did you drink last week?  1 - drinkweoQ3>=1  2 - drinkweoQ3>=2  3 - drinkweoQ3>=3  4 - drinkweoQ3>=4  5 - drinkweoQ3>=5  6 - drinkweoQ3>=6  7 - drinkweoQ3>=7  8 - drinkweoQ3>=8  >= 9 - drinkweoQ3=9 | How often do you drink five units of alcohol?  1-4 times per year - excessQ3>=2  5-10 times per year - excessQ3>=3  Once per month - excessQ3>=4  2-3 times per month - excessQ3>=5  Once per week - excessQ3>=6  2-4 times per week - excessQ3>=7  Almost every day or every day - excessQ3>=8 |
|  |  |

|  |  |
| --- | --- |
| **Information functions for all items** | **Information function across all items** |
|  |  |
| Do you drink beer? beerunQ2  Do you drink wine? wineunQ2  Do you drink liquor? spirunQ2  Have you drunk alcohol in the last year? drink12Q3  How often do you drink alcohol? alko1Q3  How many bottles of beer did you drink last week? bottweoQ3  How many glasses of wine did you drink last week? glasweoQ3  How many drinks of liquor did you drink last week? drinkweoQ3  How often do you drink five units of alcohol? excessQ3 |  |
|  |  |

|  |  |
| --- | --- |
| **Alpha reliability by score level (i.e. 1-(score standard error)^2^)** | **Test Characteristic curve (i.e. the latent variable – test score association)** |
|  |  |
|  |  |
|  |  |
